# Road traffic accidents and the contributing factors among drivers of public transportation in Mizan Aman town, Ethiopia: a Community-Based Cross-Sectional Study

**DOI:** 10.3389/fpubh.2024.1307884

**Published:** 2024-05-30

**Authors:** Mesenbet Muluken Endalew, Abraham Assefa Gibo, Mekdes Mekonen Belay, Mesfin Yimam Zegeye, Tadele Shiwito Ango, Sisay Ketema Astatke

**Affiliations:** ^1^Department of Public Health, Mizan Aman Health Science College, Mizan Aman, Southwest Ethiopia People Region, Ethiopia; ^2^Department of Nursing, College of Health Sciences, Bonga University, Bonga, Southwest Ethiopia People Region, Ethiopia; ^3^Department of Emergency Technician, Mizan Aman Health Science College, Mizan Aman, Southwest Ethiopia People Region, Ethiopia; ^4^Department of Public Health, College of Medicine and Health Science, Werabe University, Werabe, Ethiopia

**Keywords:** road traffic accidents, contributing factors, drivers, Mizan Aman town, Ethiopia

## Abstract

**Background:**

Traffic accidents on the road is an accident is a terrible accident that causes death, injury, and property damage. However, limited studies were addressed to investigate the prevalence of traffic accidents on the road and the contributing factors among drivers that help in developing strategies to cop-up the incidence within the research domain in Ethiopia, particularly in the study area.

**Objective:**

This study aimed to assess the prevalence of road traffic accidents and the contributing factors among drivers of public transportation in Mizan Aman town, Ethiopia.

**Methods:**

A community-based cross-sectional survey was employed among 376 drivers of public transportation. Every research subject was selected by using a simple random sampling technique. Semi-structured and open-ended questionnaires which comprised demographic characteristics, risky personal behaviors and lifestyles, driver’s factors, vehicle condition, and environmental conditions were used to gather data. And then after, data was collected through interviewer-administered using KoBo Collect tools. Completed data were edited and cleaned in the Kobo collect toolbox and then exported for additional analysis to a statistical tool for social science statistics version 26. The descriptive statistics were displayed as figures, tables, and texts. Binary logistic regression was analyzed to identify the contributing factors. Statistically significant was decided with a *p*-value of ≤ 0.05.

**Results:**

The results showed that the prevalence of road traffic accidents among drivers of public transportation in Mizan Aman town was 17%. The study identified factors influencing traffic accidents on the roads including marital status (being single), employee condition (permanent), monthly income (1001-2500 Ethiopia Birr), alcohol use, vehicle maintenance (not), road type (non-asphalt), and weather conditions (being windy).

**Conclusion:**

The overall prevalence of road traffic accidents among drivers of public transportation in Mizan Aman town was relatively low. Despite this, sociodemographic characteristics, driver factors, vehicle conditions, and environmental conditions [road type and weather conditions] were the predicting factors of traffic accidents in town. Therefore, reduction strategies should be the highest priority duty for concerned bodies like Mizan Aman town road and transport office, Bench Sheko zone transport and logistics office, and Southwest Ethiopia People Regional State (SWEPRS) transport bureau in the study area.

## 1 Introduction

A road traffic accident (RTA) is an incident that involves at least one moving vehicle during transportation and leaves one or more people injured or dead (1, 2). The World Health Organization (WHO) defined RTA as any injury, whether fatal or non-fatal, sustained in an accident on a public road (3). There are several scenarios in which a collision may occur (4, 5). It involves at least one moving vehicle or pedestrian, and the crash occurs on a path or street that is accessible to public transportation (3). It could result in the deaths or serious injuries of one or more people, and at least one moving car is involved (6). In most times, vulnerable road users include cyclists, motorcyclists, and pedestrians (7). If adequate measures are not taken to reduce road traffic injuries, they will become a serious public health and development concern and can damage a person’s quality of life (8). In addition to having an impact on an individual’s health, traffic accidents can also place an economic burden on households as they struggle to pay for long-term effects such as medical care, rehabilitation, and the loss of the family’s primary provider (4). Traffic accidents can also put a tremendous amount of pressure on the national healthcare systems, which already have a tragically low amount of funding (9). Furthermore, RTAs cost most nations 3% of their gross domestic product (7).

Traffic accidents continue to be a major global health and development issue, particularly in developing nations, including Ethiopia, since the road is the major route for transportation. These accidents kill close to 1.35 million people annually, ranking as the 8th most common cause of death overall, and is the leading cause of death for youth and young adults aged 5–29 years. Ethiopia has three times higher death rates due to RTAs when compared to other low-income nations (26.7 in South East Asia and 26.6 in Africa per 100,000 people) (10–12). Worldwide, approximately 90% of road deaths occur in middle-class and lower-income nations (7, 9, 13). Despite being the least motorized of the six major world regions, Africa has the highest rates of RTAs with fatality rates of 24.1/100,000 compared to the global average of 18/100,000 fatalities (14). Ethiopia reports a significant incidence of RTAs, despite having a low road network density and low automobile ownership (15). In addition, the WHO revealed a loss of approximately 400 to 500*1000,000 Ethiopian Birr (ETB) and nearly 2,000 deaths involving pedestrians (48%), passengers (45%), and drivers (7%) due to RTAs in Ethiopia (16). Furthermore, according to an Ethiopian demographic and health survey, RTAs were the second-most common accident and injury (22.8%) after unintentional falls (15).

Pieces of evidence from studies have revealed that drivers of public transportation are vulnerable to professional working groups. For instance, stressful working conditions have been associated with health and lifestyle-related outcomes among professional drivers (17). In turn, work stress in professional drivers is positively associated with traffic collisions (18). Consequently, professional drivers have suffered accidents due to work stress, and their safety has also been compromised (19). Moreover, work stress is also related to addictive behaviors such as regular alcohol consumption and smoking, which are the aggravating factors for traffic collisions among drivers (20). As a result, drivers are at more risk of suffering from muscular–skeletal, cardiovascular, gastrointestinal, and psychological problems due to specific working conditions (21).

Various prevalence of RTAs have been reported across the globe. According to a systematic research by Khatib et al., the frequency of traffic accidents resulting in injury or death varied between 11.1 and 42.6% for individuals in the 20–30 age group and between 4.6 and 97.2% for male subjects (22). Furthermore, a study conducted in Nigeria (38.3 and 35.3%) (23, 24), Vietnam (20%) (25), and Saudi Arabia (63%) (26) revealed the prevalence of RTA. Furthermore, in the national context, different studies have revealed repeated occurrences of RTA with different magnitudes. For instance, the prevalence of RTA is as follows: Hawassa (55.1%) (27); East Wollega zone (33%) (28); University of Gondar referral hospital (33.6%) (1); residents of Ethiopia (3%) (8); Amhara region (51%) (29); Wolaita zone (62.5%) (30); Bahirdar city (16.3%) (31); Chuko Town (23.5%) (32); Mekelle Town (26.4%) (33); Sidama region (55.1%) (27); Jigjiga Town (32.8%) (34); Addis Ababa (56.9%) (35); and Dilla town (39.9%) (36). Moreover, RTA causalities included drivers (21.9%), passengers (35.0%), and vulnerable road users (36.0%), of which 21.0, 12.1, and 2.9% were motorcyclists, pedestrians, and cyclists, respectively (15).

Pieces of evidence from the scientific community suggest that different contributing factors play an important role in the occurrence of RTA, which include sociodemographic characteristics and driver factors such as sex (23), age (1, 24, 27, 28), educational status (8, 24), prior punishment (28, 36), place of residence (15, 28), income status (8, 15), use of alcohol and khat (8, 24, 37, 38), personality of the driver (28), use of safety seatbelts (39), and traffic infringement (40). Stress-related working conditions (work stress, social support, and effort or reward imbalance) are relevant predictors of risky driving (41). Furthermore, risky behaviors or lifestyles of drivers were associated with driving experience, positive driving behaviors, social desirability, and perceived stress (42). Another factor that predicts traffic accidents is environmental conditions, including adverse weather conditions (37), road types (27), light conditions (43), and time of accidents (37). Other significant factors are vehicle conditions like types of vehicles (44), maintenance conditions (36), and mechanical problems.

Despite RTA being a terrible incident that causes death, injury, and property damage; there is limited information available about traffic accidents on the road and contributing factors among drivers of public transportation in developing countries, including Ethiopia. The primary sources for estimating the extent of road traffic injury in low-income countries are hospital registry data and police records; however, underreporting affects both sources (45). Previous studies addressed the prevalence of RTA among victims through a retrospective study design, which could not give significant information to decision-makers due to the design itself (45). In addition, pieces of evidence from quantitative studies revealed the prevalence of RTA (23, 28, 31, 33–36, 40, 46); in Ethiopia, particularly in the study area, there is a paucity of information regarding the contributing factors among drivers of public transportation to the occurrence of traffic accidents on the roads and public health concerns. Moreover, little was known about the prevalence of traffic accidents on the roads and the contributing factors among drivers of public transportation with a community-based cross-sectional study design. Identifying black spots and comprehending the factors contributing to traffic accidents on the roads are essential for developing remedies for public health issues (27, 47). Therefore, obtaining the magnitude and associated factors among drivers who are directly involved in the task would reveal their contribution to the occurrence of traffic accidents on the road. The study aimed to fill the gaps shown in the above pieces of literature.

The current research added a unique contribution to existing literature via a proposed approach that generated realistic findings because the study was well equipped to assess the prevalence of traffic accidents on the roads and the contributing factors among drivers of public transportation in Mizan Aman town, Southwest Ethiopia People Regional State (SWEPRS). In this regard, the rationale of the study is as follows: First, findings from the study might be used as baseline information to design effective strategies to alleviate the problems. In addition, traffic accidents are a major but mistreated public health challenge that requires determined efforts for effective and sustainable prevention; second, information from the study points out the issue regarding traffic accidents on the roads in the study area, which, in turn, primarily invites those interested volunteers to address or tackle the traffic-related problems. Moreover, information regarding the cause of traffic accidents and precautions that need to be taken by drivers was informed, promoted, and disseminated on behalf of road traffic officers; and the third and last rationale of the study was that the result obtained from the study would provide full information for readers, researchers, and policymakers to design an effective interventional strategy in the area.

### 1.1 Objectives of the study

- To determine the magnitude of traffic accidents on the road among drivers of public transportation in Mizan Aman town.
- To identify the contributing factors of traffic accidents on the road among drivers.
- To offer specific recommendations to tackle the problems based on the findings obtained from the study for responsible bodies at different levels, including Mizan Aman town, Bench Sheko zone, and Southwest Ethiopia People Region.

## 2 Methods and supplies

### 2.1 Study location, plan, and time frame

In Mizan Aman town, a community-based cross-sectional study was carried out between March and April 2023. Mizan Aman town is the capital of the Bench Sheko zone, SWEPRS. It is situated 564 kilometers away from Addis Ababa, the capital of Ethiopia. There are four Kebeles in Ethiopia, which are the smallest units of government. The total population of Mizan Aman town is 96,353, with 47,694 men and 48,659 women based on estimates from the 2007 Central Statistics Agency. Mizan Aman town is linked by an extensive road network to other cities, divisions, and regions such as Oromia and Gambela. Different interregional and regional bus transportation providers served the community of Mizan Aman town. In reality, there were 1,063 cars or vehicles registered in different unions established by the Mizan Aman town administration that provided public transportation service. Those unions include Bajaj unions (*N* = 754) in which Zenbaba (*N* = 274), Mango (*N* = 313), Aman Boeing (*N* = 167), and Taxi union (Irgib) (*N* = 55), and all vehicles were ranked from one to three based on the level of unions [Gacheb 1st rank (*N* = 53), Zihon 2nd rank (*N* = 84), and Mizan 3rd rank (*N* = 117)] (48).

### 2.2 Source and study population

All public transportation drivers found in the Mizan Aman town administration were the source population. The selected public transportation drivers were registered in the office of the Mizan-Aman town administration road transport and logistics and were considered the study population.

### 2.3 Determining the sample size and sampling methods

Using the standard formula for a single population proportion, the sample size was calculated. By taking into account 33.4% of traffic accidents on the roads due to bad road conditions in Chuko Town, Ethiopia (32), at a 95% confidence interval (CI).


$$n={\left(Z\alpha /2\right)}^{2}\left(\frac{P\;\left(1-P\right)}{d^{2}}\right)$$

$$n=\frac{{\left(1.96\right)}^{2}*0.334\left(1-0.334\right)}{\;{\left(0.05\right)}^{2}}\text{ = 342,}$$

where *n* = size of the sample; Zα/2 = 1.96 standard scores, with a 95% confidence interval; d = level margin of error to be tolerated (5%); and p = proportion of RTAs. The sample size would increase to *n* = 376 when a 10% non-response rate was taken into account.

All vehicle unions found in the Mizan Aman town were a part of the study. Information about public transport drivers and vehicles found in town was obtained from the Mizan Aman town road transport and logistics office. A total sample size was allocated to each vehicle union with their respective number of drivers being proportionate to their population size. The lists of public transport drivers in the Mizan Aman town road and transport office were the sampling frame. The entire sample size was distributed based on the real number of cars registered in the unions, namely, Bajaj unions (Mango, Zenbaba, and Aman Boeing) and Taxi union (Irgib), and all vehicles were ranked from first to third based on the level of unions (Gacheb 1st rank, Zihon 2nd rank, and Mizan 3rd rank). Then, every driver was assigned or coded with an identification number called number plate, and we entered each identification number into a database from 1 to 1,063. A random number generator was used to select 376 drivers of public transportation. Finally, a basic random selection method was used to choose each research subject. To control sampling bias in the study, we carefully defined the target population (all public transportation drivers who are serving the community in Mizan Aman town) and the sampling frame (list of drivers from the different vehicle unions from which the study subject would be drawn). In addition, the researchers made the survey accessible and short. Finally, they monitored those non-responders.

### 2.4 Inclusion and exclusion criteria

The drivers who met the following criteria were included in this study: (a) they had to be at least 18 years old; (b) they had to have been driving professionally for a minimum of a year; and (c) they had to be registered with the town’s transport and logistics office to serve the public and community through transportation.

### 2.5 Study variables

#### 2.5.1 Dependent variable

The dependent variable was traffic accidents on the roads.

#### 2.5.2 Independent variables

Demographic characteristics [sex, age, level of education, marital status, monthly income, family size, employee condition, level of driving, experience of driving, and owner of the vehicle]; risky personal behaviors [use of alcohol, khat, and cigarettes, prior punishment by traffic police, and habit of checking vehicles]; driver factors [type of vehicle he/she drives, working hours per day, information about traffic safety (media, training, friends, and traffic police), traffic-related safety training, and use of a safety belt]; vehicle conditions [types of vehicles, service of a vehicle, maintenance of vehicles, and mechanical problems]; and environmental conditions [weather conditions (rain, wind, and fog), road type (non-asphalted vs. asphalted roads), time (day, afternoon, night, and midnight), and type of collision].

### 2.6 Operational definitions

**Accident time**: This variable indicates the time during the day (24 h) when there was a high volume of traffic on the roads (49).

**Driver**: Individuals operating vehicles other than bicycles and two-wheeler vehicles (34).

**Passengers**: Any occupants of a vehicle other than the driver, including additional passengers and pillion riders.

**Pedestrians**: Individuals who use the sidewalk or streets to push, ride, or walk bicycles (34).

**Road**: Any public road system, including city streets and state, regional roadways, and local roadways (34).

**Traffic accidents on the road** include collisions or crashes involving several cars, pedestrians, animals, or physical or architectural barriers, as well as crashes that occur on a route or roadway that is accessible to the general public (3, 4, 34).

**Users of the road**: Pedestrians and all occupants of vehicles (driver, rider, and passengers) (34).

**Taxi**: A car that is driven by a professional driver and used to carry people and their small belongings across short distances (34).

**Vehicle**: Any device with three or more wheels that is used to transport people or things from one location to another (34).

**Vehicle type** refers to the various vehicle kinds, including pick-up, bus, minibus, truck, Isuzu, ambulance, car, and automobile (49).

**Driving speed**: When a driver exceeds the posted speed limit (34).

**Chewing on Khat**: A cab driver does so while operating a vehicle (34).

**Driving while intoxicated**: A cab driver who operates a vehicle after consuming alcohol within 3 h (34, 36).

**Experience of the driver**: This denotes the driver’s experience, namely, the amount of time the driver spends operating a vehicle (49).

**Vehicle service year** denotes the year the owner of the vehicle receives servicing (34, 49).

**Weather conditions** describes the weather at the time of the accident, including whether it was cloudy or foggy, windy, sunny, or wet (49).

### 2.7 Tools and methods for data collection

The tools used in this study were adapted from different published articles (27–29, 32, 34, 40) and modified to make them suitable for the study context. These tools comprise a structured and open-ended questionnaire that includes the following variables: demographic characteristics [sex, age, educational and marital status, monthly income, family size, employee condition, driving license level, the experience of the driver, and possession of a car]; risky personal behaviors [alcohol use, chewing khat, cigarette smoking, prior punishment by traffic police, and the habit of checking vehicles]; driver factors [working hours per day, information sources about traffic safety (media, training, and friends vs. traffic police), traffic-related safety training, use of a safety belt]; vehicle conditions [vehicle type, service years of a vehicle, maintenance condition, and mechanical problems encountered], and environmental conditions [weather conditions like rain, windy, fog or cloudy; road type (non-asphalted vs. asphalted); time (day, afternoon, night, and midnight), and type of collision].

Three data collectors with prior experience in data collection and who were fluent in speaking and reading in Amharic and English language were hired, and collection of the survey data underwent two-day training sessions from 14 March 2023 to 15 March 2023, on the informed consent and data collection procedures for the use of KoBoCollect.

Once the data collecting tool’s contents were finalized, Microsoft Excel was used to compute the electronic form utilizing the XLSForm standard. Range, checks for logic, and rationale for skipping were incorporated into the electronic form. To verify syntax, XLSForm Online v2.x was used. After the verification, the XLSForm was uploaded via the KoBo Toolbox website. With its web-based form builder, KoBo Toolbox also facilitates form design. Throughout the entire data collection period, we allocated each of the three Android devices running KoBoCollect version 2022.2.3 to a single data collector. A Google account and a KoBo account were created for each smartphone to link the data collected to a particular smartphone and data collector. Each data collector downloaded the blank form to their smartphone from the KoBo Toolbox server. The completed form data were transferred from the smartphones to the server (administer account) after each day of data collection. In conclusion, the technology provides an additional degree of protection by prohibiting smartphones from accessing the aggregated collected data from the server (50).

### 2.8 Data quality assurance

Professionals with linguistic backgrounds who hold a Bachelor of Arts (BA) degree in Amharic translated the tools from English into the national language, Amharic, and then backtranslated to English for accuracy. Tools were adapted from published articles (27–29, 32, 34, 40) and were pretested among 5% (19 study participants) in Bonga Town, Kaffa Zone, before data collection, and any corrections made after the pre-test or not were stated. To guarantee the accuracy, consistency, and completeness of the data collected, data collectors received orientation and rigorous oversight. The consistency and completeness of the collected data were verified. Before analysis, a first analysis was performed.

### 2.9 Data processing, analysis, and interpretation

The completed aggregate of collected data from the server was downloaded, edited, and cleaned. After that, it was exported to Statistical Package for the Social Sciences, version 26 to be further examined. Frequency and percentage were used to summarize descriptive data presented in texts, tables, and figures. To evaluate the contributing factors, logistic regression analysis was performed. To control the impact of confounders, multivariable logistic regression was performed for the variables in the bivariable logistic regression that had a *p*-value of ≤ 0.25. Hosmer and Lemeshow statistical tests were carried out to check the goodness of fit. The variables were selected using a backward stepwise regression (LR) method. The degree of the connection was assessed by computing the adjusted odds ratio (AOR) with 95% confidence intervals. For variables in the final models, *p*-values less than 0.05 denote statistical significance.

### 2.10 Ethical considerations and participant consent

The study was carried out following ethical approval granted by the institutional review committee of the Mizan Aman Health Science College under reference no: PN003/2023. Furthermore, informed consent was requested from the relevant entity verbally and in writing. The individuals’ privacy and the overall information collected from them were kept completely confidential by using codes. Regarding ethical issues, the voluntary participation of key informants was respected, informed consent was obtained, and no harm to human beings was addressed. To stop the spread of COVID-19, personal protective equipment such as masks was used.

## 3 Results

### 3.1 Sociodemographic characteristics

**Figure 1:**
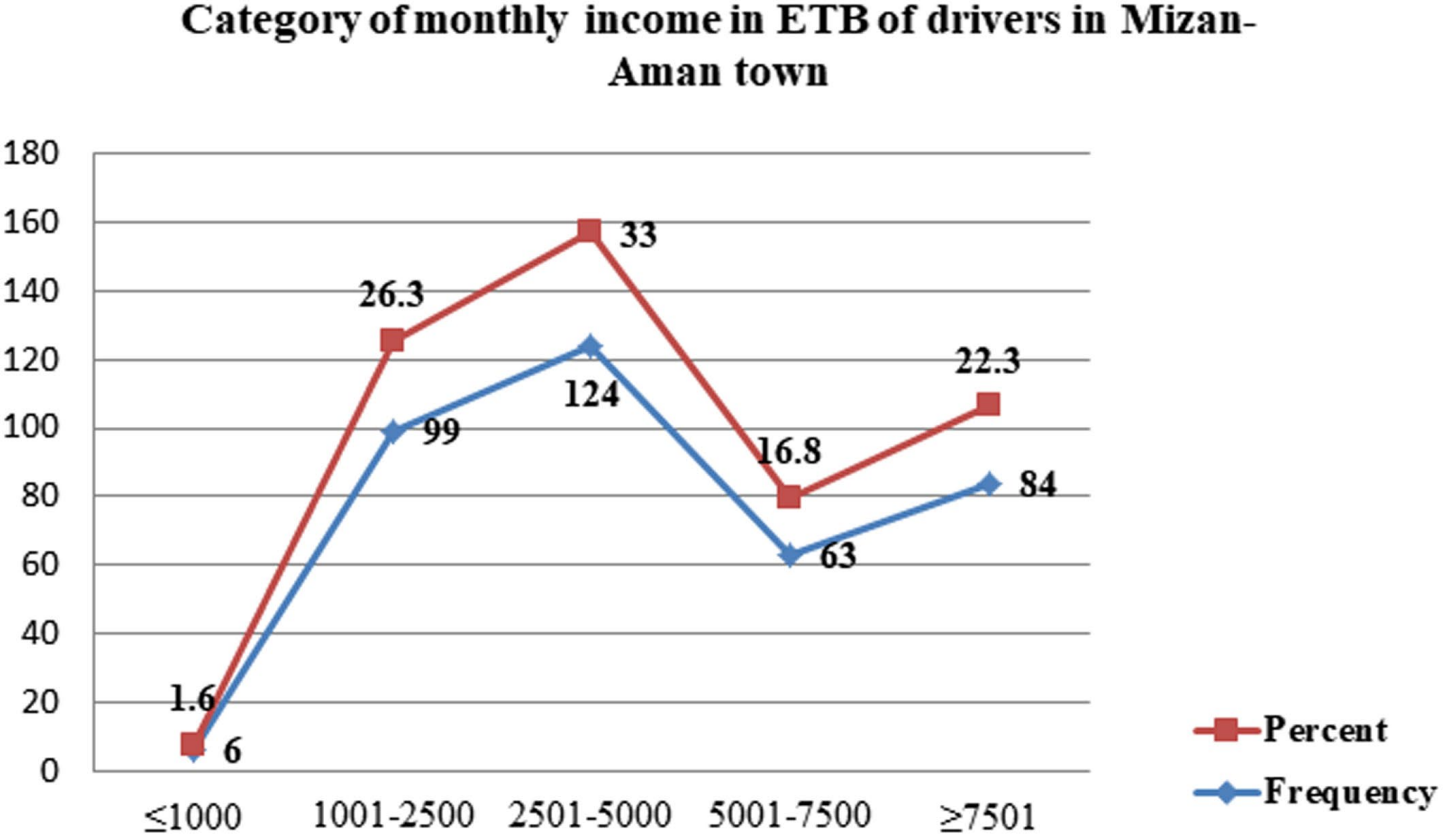
Monthly income category among drivers in the Mizan-Aman town, Bench Sheko zone, SWEPRS, Ethiopia, 2023 (*n* = 376).

A total of 376 people in all, or 100% of the respondents, took part in the research. Nearly 371 (98.7%) study participants were men. The mean age of the study participants was 28.12 ± SD of (6.322). Nearly 227 (60.4%) and 149 (39.6%) of the study participants were married and single. More than three-quarters of the respondents 326 (86.7%) attended secondary school. In terms of working conditions, 255 (67.8%) of the study participants were employed permanently. In addition, the majority of drivers (257, 68.4%) have fewer than 5 years of experience. A total of 359 (95.5%) drivers had less than or equal to five family members. The mean monthly income of respondents was 4859.04 ± SD of (2733.149) ETB (Table 1). The majority, 124 (33%), of drivers reported that they have a monthly income range of 2,501–5,000 ETB (Figure 1).

**Table 1:** Socio-demographic characteristics of drivers of public transportation in Mizan Aman town, Bench Sheko zone, SWEPRS, Ethiopia, 2023 (*n* = 376).

Variables	Category	Frequency	Percent
Sex	Male	371	98.7
	Female	5	1.30
Age in years	18–24	124	33.0
	25–34	189	50.3
	35–44	54	14.4
	≥45	9	2.4
	Mean	28.12 ± SD of (6.322)	
Marital status	Single	149	39.6
	Married	227	60.4
Educational status	Attended primary school	37	9.8
	Attended secondary school	326	86.7
	Attended college and above	13	3.50
Employee condition	Permanent	255	67.8
	Part time	121	32.2
Driving experience in years	<5	257	68.4
	5–10	83	22.1
	>10	36	9.60
Level of driving license	Taxi	146	38.8
	Public-1	155	41.2
	Public-2	75	19.9
Family size	≤5	359	95.5
	>5	17	4.50

### 3.2 Drivers factors and vehicles conditions

In this study, nearly 373 (99.2%) of the respondents replied that they received traffic-related safety training, of which approximately 316 (84.0%) engaged in traffic-related safety training before starting a job. Accordingly, approximately 372 (98.9%) drivers replied that they have a habit of checking vehicles. Of which 333 (88.6%) reported that they have a habit of checking their vehicle in the morning. More than half, 193 (51.3%), of the drivers use a safety belt while driving, of which 157 (41.8%) reported that they always use a safety belt (Table 2).

**Table 2:** Driver’s factors and vehicle conditions related with the prevalence of traffic accidents on the roads among drivers of public transportation in Mizan Aman town, Bench Sheko zone, SWEPRS, Ethiopia, 2023 (*n* = 376).

Variables	Category	Frequency	Percent
**I. Driver’s factors**			
Working hours per day	≤8 h	118	31.4
	>8 h	258	68.6
Traffic-related safety training	Yes	373	99.2
	No	3	0.80
Engaged in traffic-related safety training	Before starting job	316	84.0
	After engaged job	57	15.2
The habit of checking vehicles	Yes	372	98.9
	No	4	1.10
Time of checking vehicles	At the morning	333	88.6
	Throughout the day	39	10.4
Use a safety belt	Yes	193	51.3
	No	183	48.7
Frequency of safety belt use	Always	157	81.3
	Usually	2	1.00
	Sometimes	34	17.6
Drive above the recommended speed	Yes	309	82.2
	No	67	17.8
**II. Vehicle conditions**			
Type of vehicle	Bajaj	180	47.9
	Minibus/Midbus	117	31.1
	Bus	79	21.0
Service of years of a vehicle	1–4	290	77.1
	5–9	75	19.9
	10–14	8	2.10
	≥15	3	0.80
Owner of the vehicle	Self	46	12.2
	Family	125	33.2
	Others	205	54.5
Maintenance of vehicles	Yes	191	50.8
	No	185	49.2
Mechanical problems encountered in vehicles	Yes	146	38.8
	No	230	61.2

In the present study, approximately 180 (27.9%) and 117 (31.1%) Bajaj and Taxi/Dolphin types of vehicles were the most public transport serving the community, respectively. Of those, approximately 290 (77.1%) of the vehicles’ service years were of category 1–4 years. Furthermore, nearly half of the vehicles, 185 (49.2), serving the public in the study area have not been maintained in recent years. Similarly, approximately 230 (61.2%) respondents reported their vehicles were encountered with mechanical problems (Table 2).

**Figure 2:**
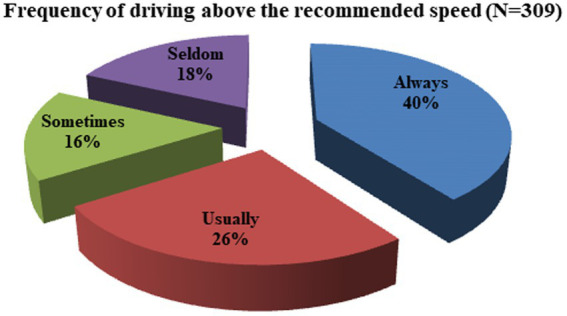
Frequency of driving above the recommended speed among divers in the Mizan-Aman town, Bench Sheko Zone, SWEPRS, Ethiopia, 2023.

**Figure 3:**
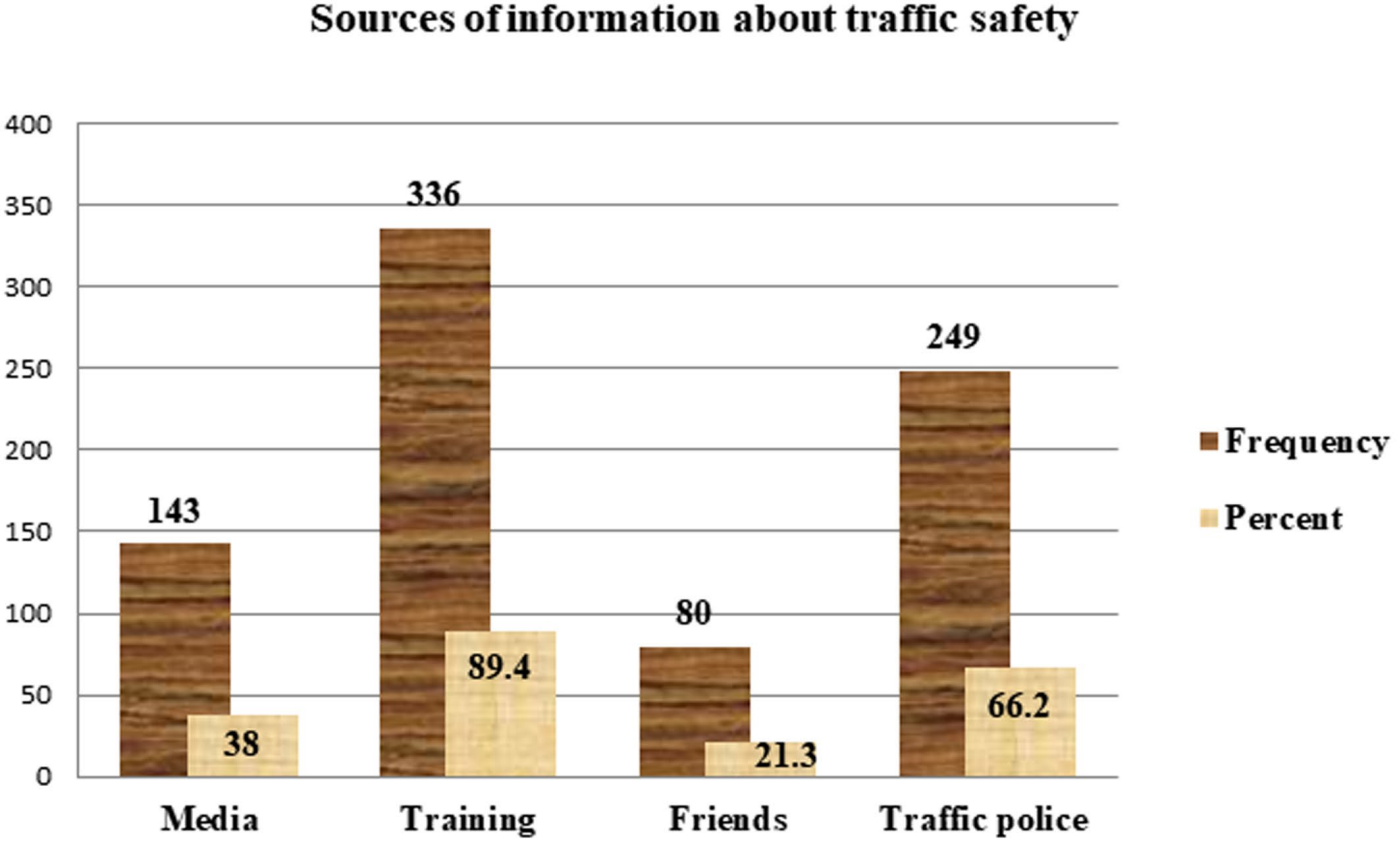
The sources of information about traffic safety among divers in the Mizan-Aman town, Bench Sheko Zone, SWEPRS, Ethiopia, 2023.

In addition, 309 (82.2%) drivers have the habit of driving above the recommended speed (Table 2). The frequency of driving above the recommended speed among those drivers was always (40%), usually (26%), seldom (18%), and sometimes (16%) (Figure 2). The major sources of information about traffic safety for the study participants were training (89.4%) and traffic police (66.2%) (Figure 3).

### 3.3 Risky personal characteristics

**Figure 4:**
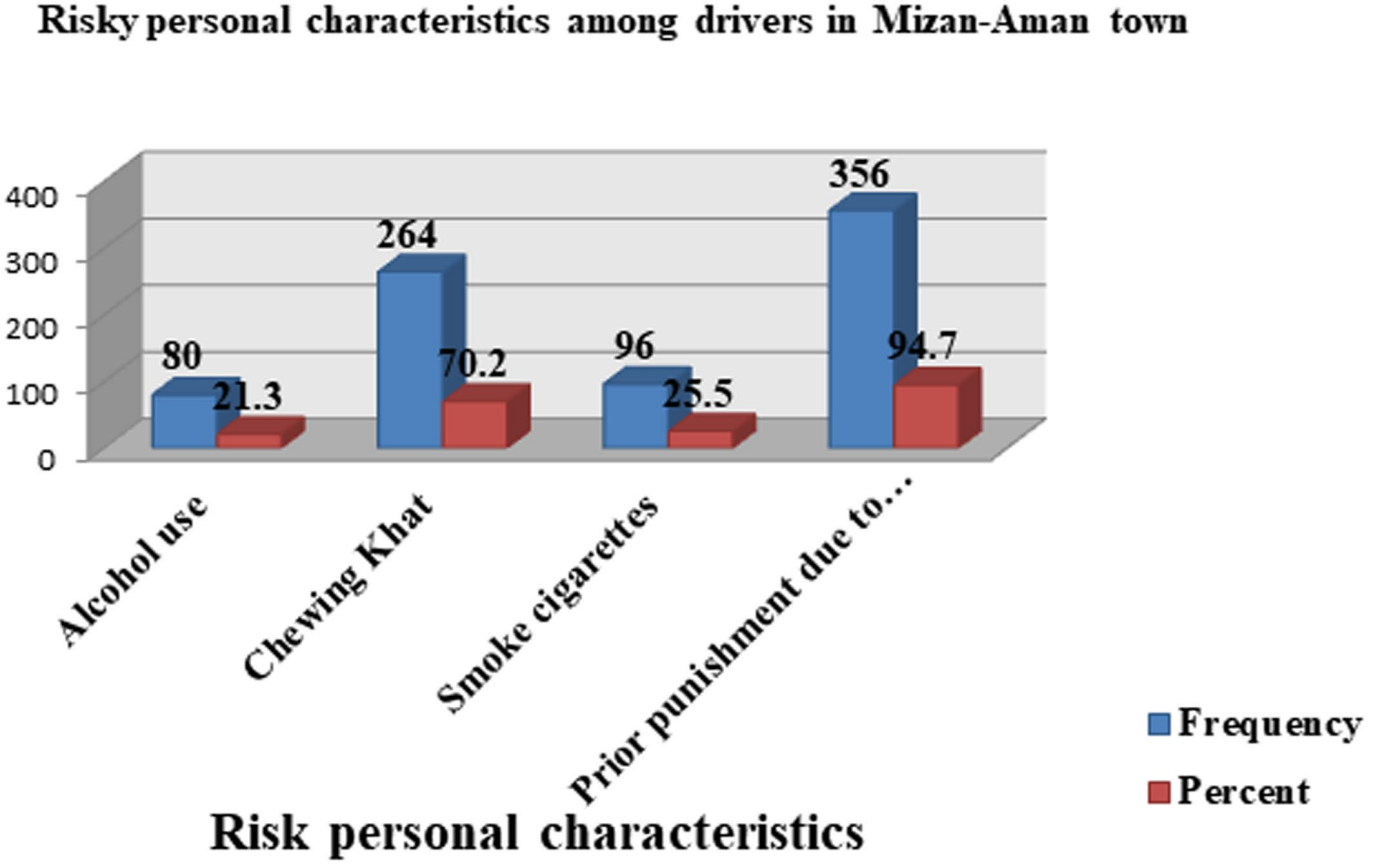
Risky personal characteristics among drivers in the Mizan-Aman town, Bench Sheko zone, SWEPRS, Ethiopia, 2023 (*n* = 376).

**Figure 5:**
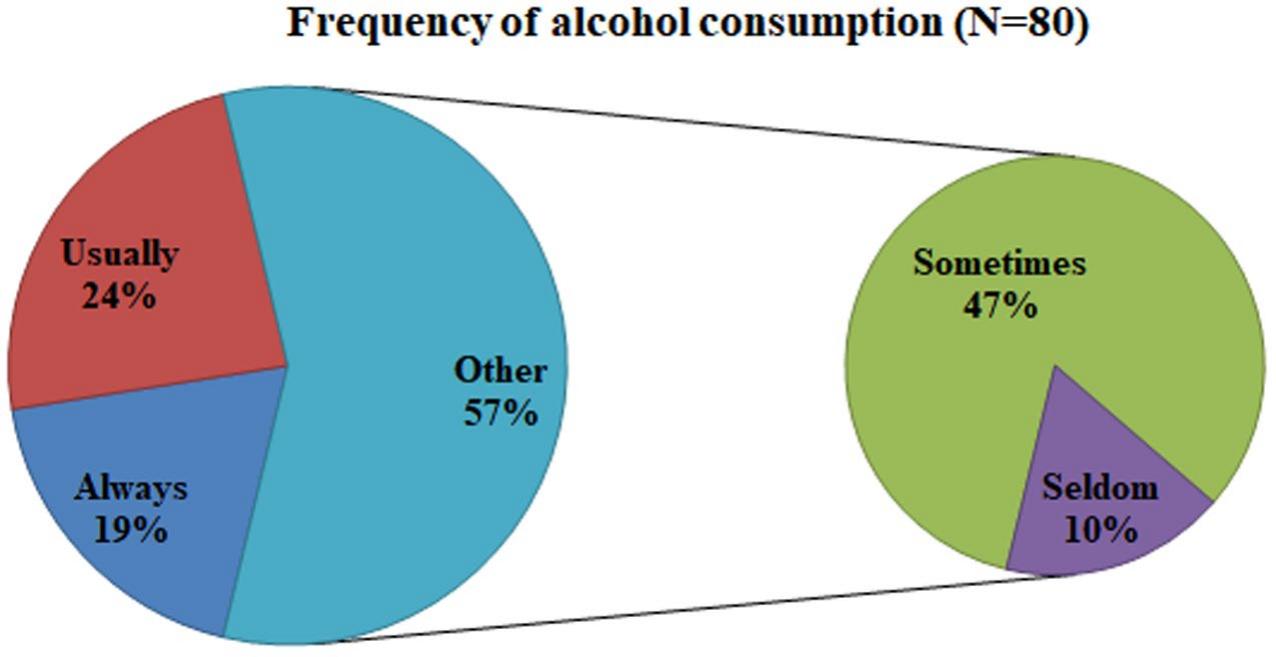
Frequency of alcohol use among drivers in the Mizan-Aman town, Bench Sheko zone, SWEPRS, Ethiopia, 2023.

**Figure 6:**
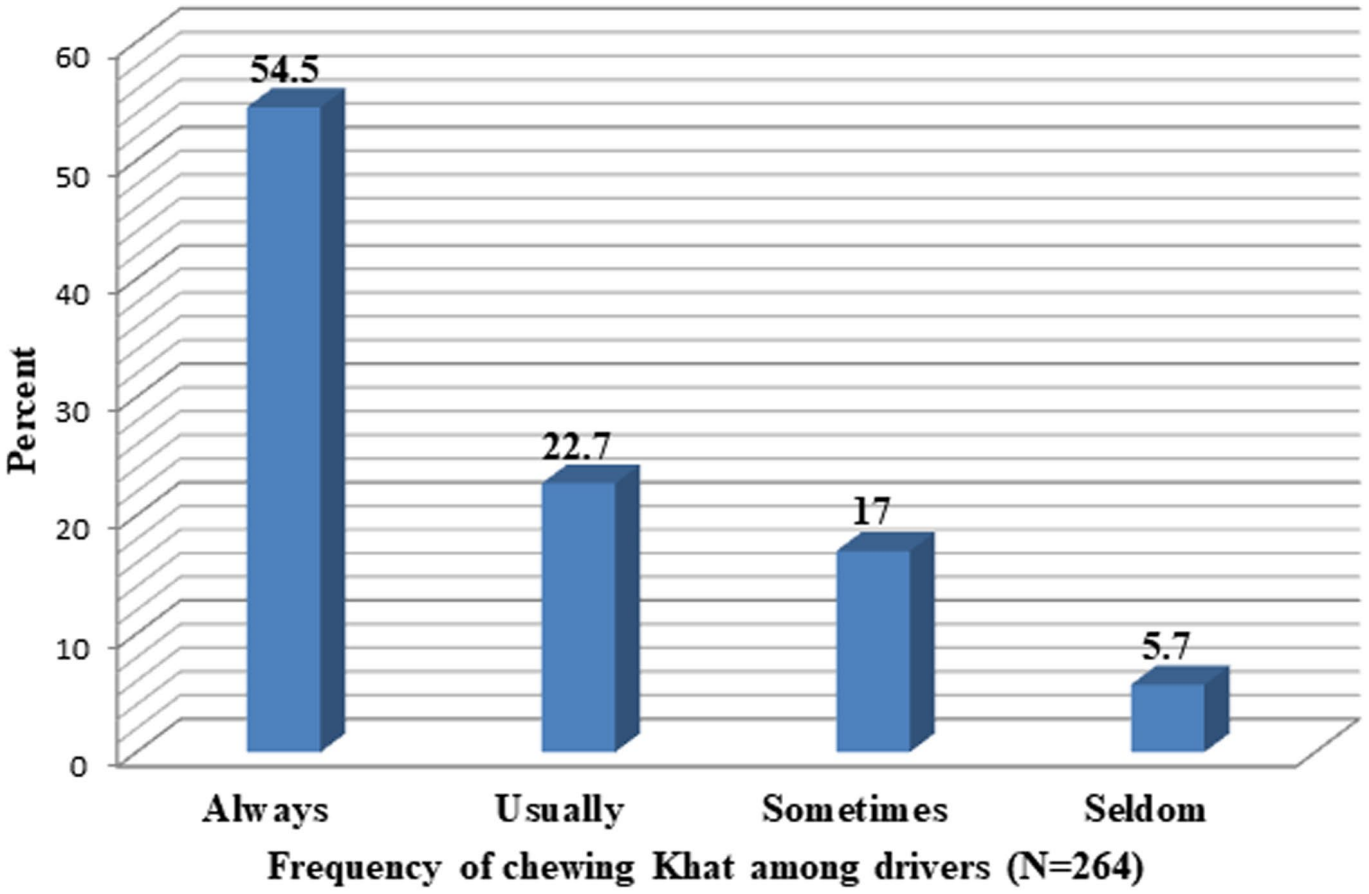
Frequency of chewing Khat among drivers in the Mizan-Aman town, Bench Sheko zone, SWEPRS, Ethiopia, 2023.

Regarding addiction-related risk factors, approximately 80 (21.3%) and 264 (70.2%) drivers were alcohol users and khat chewers, respectively, in this study. The majority of drivers [356 (94.7%)] were punished by traffic police for disregarding traffic rules (Figure 4). Moreover, the frequency of alcohol consumption/use among drivers shown below (Figure 5) was sometimes (47%), usually (24%), always (19%), and seldom (10%) whereas the frequency of chewing Khat among drivers shown below (Figure 6) were always (54.5%), usually (22.7%), sometimes (17%), and seldom (5.7%) in the study area.

### 3.4 Prevalence of traffic accidents on the road among drivers

**Figure 7:**
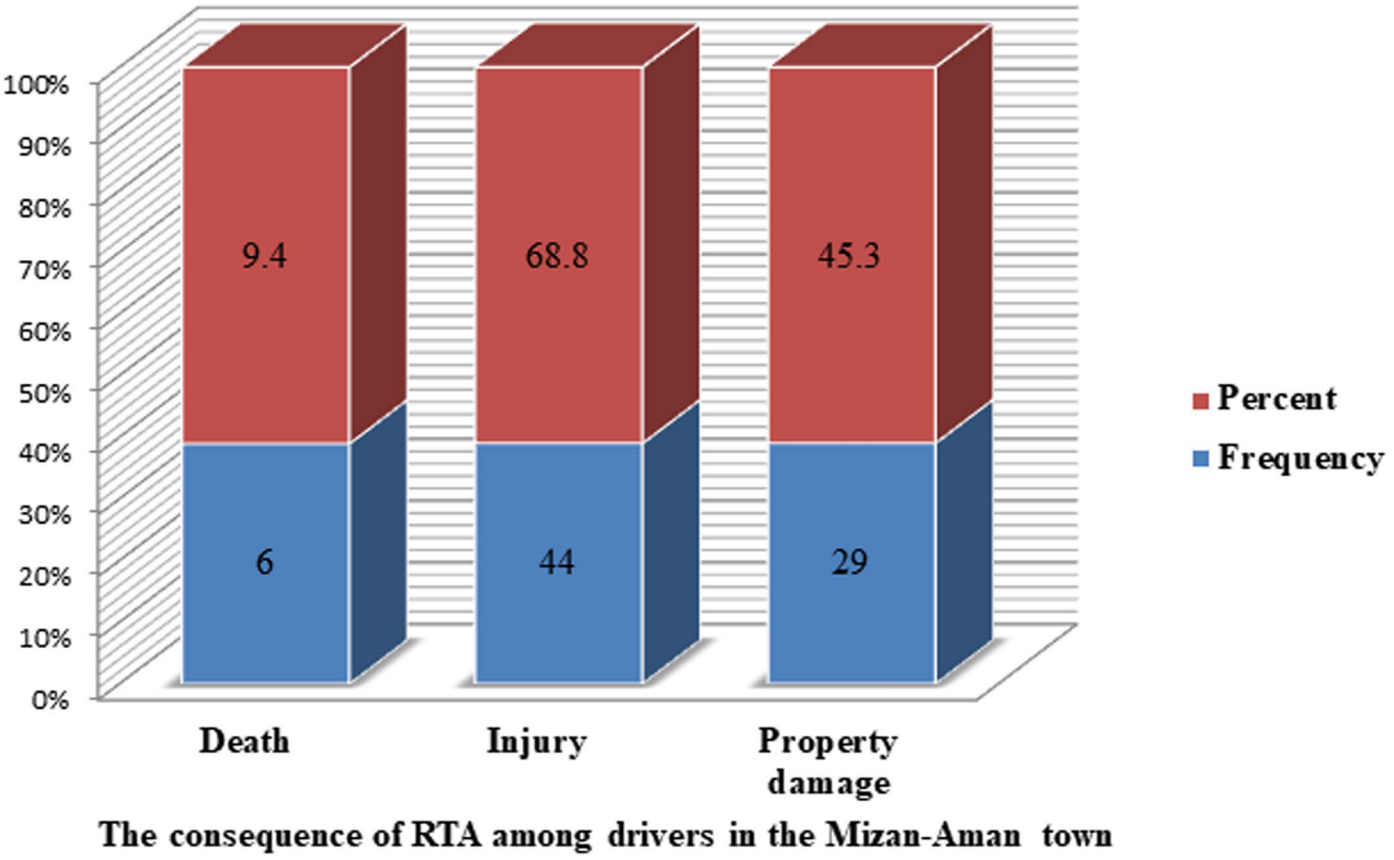
The consequence of RTA in the last 3 years among drivers in the Mizan-Aman town, Bench Sheko zone, SWEPRS, Ethiopia, 2023.

**Figure 8:**
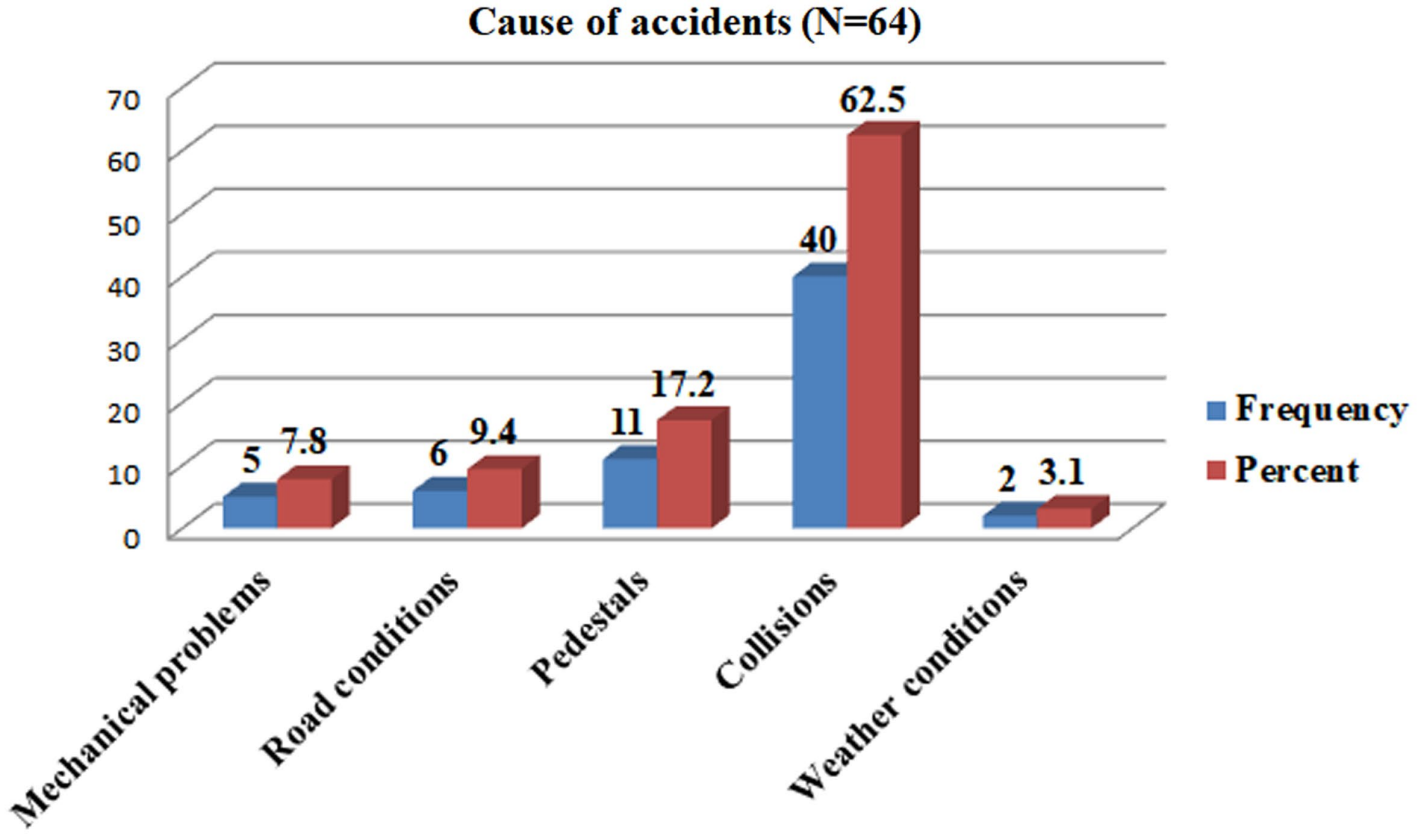
The reasons for the occurrence of RTA in the last 3 years among drivers in the Mizan-Aman town, Bench Sheko zone, SWEPRS, Ethiopia, 2023.

The prevalence of traffic accidents on the road among drivers of public transportation in the last 3 years was 17% (95% confidence interval: 13.0–21.0) (Table 3). The resulting consequences were injury 44 (68.8%), property damage 29 (45.3%), and death 6 (9.40%) (Figure 7). Most of the accidents happened in the afternoon 37 (57.8%) and morning 26 (40.6%). The majority, 56 (87.5%), of the reported accidents happened on asphalt, whereas 33 (51.6%) occurred during windy weather conditions (Table 3). The major root causes for the occurrence of reported accidents were collisions, pedestrians, road conditions, mechanical problems, and weather conditions 40 (62.5%), 11 (17.2%), 6 (9.4%), 5 (7.8%), and 2 (3.10%) (Figure 8).

**Table 3:** Prevalence of traffic accidents on the roads among drivers of public transportation in Mizan Aman town, Bench Sheko zone, SWEPRS, Ethiopia, 2023.

Study variables	Category	Frequency	Percent
Traffic accidents on the roads in the last 3 years	Yes	64	17.0
	No	312	83.0
If the cause of the accident is a collision, the type of collision (*N* = 40)	Head-on collision	28	70.0
	Rear-ended	11	27.5
	Rear-ended a vehicle in front of you	1	2.5
Injured happened on whom	Passenger	19	29.7
	Pedestrian	33	51.6
	Driver	17	26.6
The death happened on whom	Passenger	2	33.3
	Pedestrian	4	66.7
Place of accident	In the city	35	54.7
	Outside of city	29	45.3
Weather conditions during the accident	Rain	24	37.5
	Windy	33	51.6
	Fog	11	17.2
The road type where the accident happened	Asphalt	56	87.5
	No asphalt	8	12.5
A time when the accident happened	Morning	26	40.6
	After noon	37	57.8
	Night	1	1.6

### 3.5 Factors associated with the prevalence of traffic accidents on the road

In bivariable logistic regression, variables including marital status, employee condition, monthly income, engagement in traffic-related safety training, chewing Khat, maintenance of a vehicle, vehicle encountered with mechanical problems, road type, and weather conditions were significant. In the multivariable logistic analyses, marital status, employee condition, monthly income, alcohol use, vehicle maintenance, road type, and weather conditions were found to be significantly associated with traffic accidents on the roads at a significance level of *p* = 0.05 (Table 4).

**Table 4:** Factors associated with traffic accidents on the roads among drivers of public transportation in Mizan Aman town, Bench Sheko zone, SWEPRS, Ethiopia, 2023 (*n* = 376).

Study variables	Category/response	Prevalence of traffic accidents on the roads		COR (95% CI)	AOR (95% CI)	*p*-value
		Yes	No			
Age in years	18–24	18	106	2.944 (0.675, 12.847)	0.137 (0.015, 1.278)	0.081
	25–34	32	157	2.453 (0.583, 10.324)	0.274 (0.032, 2.361)	0.239
	35–44	11	43	1.955 (0.421, 9.081)	0.514 (0.057, 4.616)	0.552
	≥45	3	6	1	1	1
Marital status	Single	17	132	2.027 (1.114, 3.689)	0.448 (0.202,0.994)	0.048*
	Married	47	180	1	1	1
Employee condition	Permanent	26	229	4.032 (2.307, 7.049)	3.343 (1.587, 7.041)	0.001*
	Part time	38	83	1	1	1
Monthly income in Ethiopian Birr	≤1,000	1	5	1.087 (0.118, 9.992)	0.616 (0.032,12.006)	0.749
	1,001–2,500	24	75	0.679 (0.330, 1.400)	0.377 (0.143, 0.992)	0.048*
	2,501–5,000	10	114	2.478 (1.055, 5.823)	2.271 (0.773, 6.672)	0.136
	5,001–7,500	14	49	0.761 (0.337, 1.719)	0.483 (0.171, 1.363)	0.169
	≥7,501	15	69	1	1	1
Family size	≤5	63	296	1	1	1
	>5	1	16	0.294 (0.038, 2.255)	–	–
Working hours per day	≤8 h	16	102	1	1	1
	>8 h	48	210	0.686 (0.372, 1.267)	–	–
Traffic-related safety training	Yes	62	311	1	1	1
	No	2	1	0.100 (0.009, 1.116)	–	–
Engaged in traffic-related safety training	Before starting job	61	283	1	1	1
	After engaged job	1	28	6.035 (0.806, 45.213)	–	–
Alcohol use	Yes	10	70	1.562 (0.756, 3.226)	4.083 (1.415,11.780)	0.009*
	No	54	242		1	1
Chewing Khat	Yes	52	212	0.489 (0.250, 0.957)	–	–
	No	12	100	1	1	1
Drive above the recommended speed	Yes	49	260	1.531 (0.799, 2.933)	–	–
	No	15	52	1	1	1
Maintenance of vehicles	Yes	41	150	1	1	1
	No	23	162	1.925 (1.103, 3.360)	3.250 (1.483, 7.120)	0.003*
Mechanical problems encountered on the vehicles	Yes	36	110	0.424 (0.245, 0.731)	–	–
	No	28	202	1	1	1
Road type	Asphalt	30	104	1	1	1
	Not asphalt	34	208	1.765 (1.024, 3.042)	3.222 (1.400, 7.414)	0.006*
Weather conditions	Rain	21	200	1.323 (0.594, 2.943)	1.325 (0.516, 3.402)	0.558
	Windy	33	40	0.168 (0.075, 0.377)	0.104 (0.038, 0.284)	0.000**
	Fog (cloudy)	10	72	1	1	1

COR, Crude Odd Ratio; AOR, Adjusted Odd Ratio; 1: Reference category. *: Statistically significant at *p* < 0.05, **: Statistically significant at *p* = 0.000.

Compared to part-time workers, drivers with permanent jobs had a three times higher risk of being involved in traffic accidents on the roads (AOR = 3.343; 95% CI: 1.587, 7.041). Drivers who consumed alcohol had four times higher odds of being involved in traffic accidents on the roads than those who did not (AOR = 4.083; 95% CI: 1.415, 11.780). Additionally, according to this study, drivers who operate poorly maintained vehicles are almost three times more likely than their peers to be involved in traffic accidents on the road (AOR = 3.250; 95% CI: 1.483, 7.120). Drivers who had driven on non-asphalted road types had three times higher odds of being involved in traffic accidents on the road than drivers who had driven on asphalted road types (AOR = 3.222; 95% CI: 1.400, 7.414). Drivers who have experienced driving in windy weather conditions were 89.6% less likely to suffer from traffic accidents on the roads than drivers who experienced driving in foggy weather conditions (AOR = 0.104; 95% CI: 0.038, 0.284) (Table 4).

## 4 Discussion

This is the first study that has assessed the prevalence of traffic accidents on the roads and contributing factors among drivers of public transportation in Mizan Aman town, Bench Sheko zone, SWEPR, Ethiopia.

In the current study, the prevalence of traffic accidents on the roads among drivers of public transportation in the past 3 years was found to be 17%. This result is consistent with results reported in previous studies conducted in different countries: Ibadan, Nigeria (16.2%) (46), Hanoi, Vietnam (20%) (25), and Ethiopia [Bahirdar city and Amhara national regional state] (16.3 and 20%) (31, 51). The main reasons for the occurrence of the accidents might be due to stressful working conditions (17), job stress (18), safety compromised by job stress (19), job stress related to addictive behaviors (regular alcohol consumption and smoking) (20), and psychological problems (anxiety, depression, and post-traumatic stress disorder) (21). Implementation of strategies to combat RTAs includes road safety training courses for young people and adults aimed at raising awareness and changing the attitudes of their participants, influencing the intention to change behaviors toward more appropriate and less risk factors among drivers, and evaluations of different types of programs regarding road traffic, which specifically focused on resilience, risk detection, and decision-making that have proven to be the most useful (52).

However, the result is somewhat lower than the study results reported in Mekelle Town (26.4%) (33), Chuko Town (23.5%) (32), and Ethiopia (21.9%) (15). The observed variation may be attributed to variations in the data sources, study participants, study settings, and sample size (15, 32, 33). The other reason that caused the variation might be the source of obtained information related to the occurrence of traffic accidents on the roads in the form of questionnaires in which some respondents purposely close or hide some important information. On the other hand, the figure is higher than the result reported from an Ethiopian study from 2015 using the STEPS survey, in which 3% of the participants had been in a traffic accident (8). This significant disparity may result from variations in the study population, the study period and conditions, the sampling strategy, methods for analyzing descriptive data, and sociodemographic traits. Moreover, the result is lower than what is reported in different countries having a history of traffic accidents on the roads in the past years: Bauchi State, Nigeria (38.3%) (23), Hail, Saudi (63%) (26), and Ethiopia [Sidama region, Jigjiga town, Nekemte town, Dilla town, and Addis Ababa] (55.1, 32.8,33, 39.9, and 56.9%) (27, 28, 34–36). The difference might be due to differences in sample sizes such as of Addis Ababa (840), East Wollega Zone (400), and Saudi Arabia (208), respectively (26, 28, 35). In addition, the reasons for the difference could be because of a large population or over-crowdedness in previous studies (23, 34, 35) and the environments for study, the research subjects and design, the technique of sampling, and the data source (27).

This study’s findings show that sociodemographic attributes of public transport drivers were strongly correlated with the occurrence of traffic accidents on the roads in the town. Regarding marital status, those drivers who were single were 56% less likely to be involved in the occurrence of traffic accidents than drivers who were married. This might be because married drivers were responsible for taking care of their families as the only source of income, so they run timelessly for extra income, which leads them to be more involved in the occurrence of traffic accidents on the roads. Moreover, a study conducted in Vietnam (40) and another in Dilla Town (36) showed that taxi drivers who had been married were not significantly involved in road traffic accidents. This may be the result of variations in the sample population and the type of vehicle involved in the study. In other words, the study conducted in Vietnam was limited to taxi drivers (40), but in this study, all public transport service providers were included.

Compared to part-time workers, drivers with permanent jobs had a three times higher risk of being involved in traffic accidents. Risk is the probability of getting harm (injury, illness, death, damage, etc.) that may occur from exposure to a hazard. In this scenario, drivers being full-time employed were more exposed to hazards, which might lead them to be involved in RTA. In addition, permanent drivers may be under social and economic pressure because of inflation. This finding was inconsistent with a study result reported in Vietnam in which taxi drivers employed as part-time were 2.22 times more likely to be involved in RTA than drivers employed full-time (40). In addition, a study carried out in Nigeria among drivers employed by state institutions revealed that drivers with part-time occupations were 2.6 times more likely to be involved in RTA than those without such professions (46). This discrepancy might be due to the additional need for income sources because of high expenditure on social needs such as education and health and economic competition driven by the need of drivers who were employed part-time in Vietnam (40). Since the RTA is a sudden phenomenon, full-time drivers were more exposed to RTA than part-time drivers.

This study finding showed that drivers with a monthly income of 1,000–2,500 ETB were 63% less likely to be encountered in RTA than those below 1,000 ETB and above 2,500 ETB. This result is in line with those of studies conducted in Vietnam and Ethiopia, which disclosed that inadequate revenue collection was strongly linked to RTA, and households with annual incomes exceeding 30,000 ETB were the most engaged in RTA (8, 40). In addition, the result is also in line with that of a study conducted in Addis Ababa, Ethiopia (38). The research carried out in the Amhara region revealed that being wealthy and in the middle-income strata were 8 and 40% less likely to be involved in the occurrence of RTI, respectively (51). However, the findings in Dilla Town among drivers were not significant with respect to RTA (36). This discrepancy may be brought about by the sociodemographic traits of the research subjects in the study region. It is supported by a qualitative finding that showed that economic pressure on the safety of drivers had contributed to RTA (53).

In fact, the use of alcohol is one of the risky personal behaviors that expose drivers to traffic accidents on the roads. A study conducted in North Gondar disclosed that driver’s behavior contributes (67%) more than other factors in causing RTA (54). Similarly, an Iranian study found that the primary risk factors for the incidence of RTAs were the actions of drivers inside the transportation system (55). The results of this study showed that the likelihood of experiencing a traffic collision on the roads was 4.08 times higher for those who used or consumed alcohol. This result is consistent with previous study results reported in different countries: Ibadan, Nigeria (24), Ethiopia, Addis Ababa (38), and Mekelle town (33). It is further bolstered by another research study carried out in Dilla town, which showed that involvement in traffic accidents was over eight times higher among drivers who exhibited risky driving behaviors compared to those who did not (36). Despite this finding, a result reported in the United States showed that alcohol consumption as a cause of RTA causalities was less likely (56). This disparity may result from variations in the research settings, the sociodemographic makeup of the populations, transportation laws or policies among the nations. Similarly, a study conducted in Eastern Wollega and Vietnam showed that the habit of alcohol consumption was not significant for the occurrence of RTA (28, 40). This could be because of variations in the research area or environments, the sociodemographic makeup, transportation laws, or policies among the nations.

This study found that drivers who operate poorly maintained vehicles are almost three times more likely than their peers to be involved in traffic accidents on the road. This result is corroborated by those reported in Vietnam (28) and Dilla Town, Ethiopia (36).

Road conditions play a pivotal role in the occurrence of traffic accidents on the road. Drivers who had driven on non-asphalted road types had three times higher odds of being involved in traffic accidents on the road than drivers who had driven on asphalted road types. This result aligns with a result reported in Hawassa, Ethiopia (27), which might be due to similarities in the geography of the study area. The road features are, in turn, determined by natural topography, the quality of roads by themselves, the absence of roadside traffic signals, and the absence of speed breakers, especially on sloppy roads.

The likelihood of traffic accidents occurring on the roads was 89.6% lower when windy weather prevailed compared to rain, fog, and overcast conditions. This might be because the weather conditions of the study area, particularly SWEPRS, were most likely rain, fog, and overcast conditions, rather than windy. The finding aligns with that of research conducted at the Burayu town police stations in Ethiopia, which indicated rainy weather conditions that caused the fatality in RTAs (39). It is also supported by findings reported in the United States (56). A review of factors that cause RTA in Africa identified that environmental factors cause one-fourth of the accidents in the region (57). According to a systematic review conducted by Jakobsen et al., external factors were found to have an influence on occupational risk factors for road traffic crashes among professional drivers (58). Moreover, a study carried out in Pakistan showed weather conditions such as rain, wind, and fog as a determinant factor for the occurrence of traffic accidents (59). A similar finding in Iran indicated that unsupportive environmental conditions contribute to RTAs (60). This inference is reinforced by the findings of a qualitative study conducted in an industrial city in India, which demonstrated that inclement weather not only exacerbates poor road conditions but also reduces visibility for motorists traveling in the opposite direction (61). However, Dajun Dai revealed that weather conditions were not significant risk factors for the occurrence of RTA (43).

Despite these predicting factors such as age (24, 27, 28, 34, 46); driving above the recommended speed (27, 33); mechanical problems encountered on the vehicle (33); chewing khat (8, 34), and attending traffic safety-related training (36) investigated in previous studies, there is no observed statistical association with RTA in the current study.

### 4.1 Limitations of the study

First, there was no control over characteristics that would have limited the ability to draw conclusions about causality or advocate policies based on the findings because the study participants had access to all pertinent information. Second, bias because of the study’s design relied on interviewers to get self-reported data and information from participants; these data may be influenced by social desirability or recollection bias. Third, limited availability of data – data obtained from previous studies, reports, or databases may not provide the most updated or comprehensive information about traffic accidents on the roads – may limit the accuracy and generalization of the findings. Finally, there was lack of qualitative data, which should be incorporated into future studies.

## 5 Conclusion

Incidence of traffic collision on the road among drivers of public transportation in Mizan-Aman town is 17%. Some predictive factors were found to be associated with the likelihood of traffic accidents, including weather, road type, marital status, alcohol consumption, and car maintenance.

Therefore, implementation of reduction strategies for RTAs like speed breakers, alcohol tracing, and strong traffic safety guidelines and laws ought to be the highest priority for concerned bodies such as the regional transport office, zonal transport office, and road safety and traffic police to mitigate these problems in the study area. In addition, enhancing awareness about road safety rules and regulations should be given to drivers as well as to the community.

## Data availability statement

The original contributions presented in the study are included in the article/Supplementary material, further inquiries can be directed to the corresponding author.

## Ethics statement

The study was conducted after ethical clearance was approved with reference PN003/2023 by the institutional review committee of Mizan Aman Health Science College. Furthermore; both oral and written informed consent were sought from the concerned body. The overall information obtained from the study participants and their privacy was kept strictly confidential using codes.

## Author contributions

ME: Conceptualization, Data curation, Methodology, Writing – original draft. AG: Writing – original draft, Writing – review & editing. TA: Conceptualization, Data curation, Formal analysis, Funding acquisition, Investigation, Methodology, Project administration, Resources, Software, Supervision, Validation, Visualization, Writing – original draft, Writing – review & editing. SKA: Conceptualization, Data curation, Formal analysis, Investigation, Methodology, Software, Supervision, Validation, Visualization, Writing – original draft, Writing – review & editing. MB: Writing – original draft, Writing – review & editing. MZ: Writing – original draft, Writing – review & editing.
