# Supplementary material for: Road traffic accidents and the contributing factors among drivers of public transportation in Mizan Aman town, Ethiopia: a Community-Based Cross-Sectional Study
Source: Front Public Health. 2024 May 30;12:1307884. doi: 10.3389/fpubh.2024.1307884 (PMC11192049; doi:10.3389/fpubh.2024.1307884)
Supplement: Supplementary file 1 [file Data_Sheet_1.PDF]

Semi-structured Questionnaires

Date_________, Participant's code_________, address ___________

Direction: Mark "√" in the box for the given answers to the questions

**No**	**Questions**	**Category**
**Part I: Socio-demographic characteristics of the participants (drivers)**		
1.	Sex	1. Male<br>2. Female
2.	Age of respondent	___________years
3.	What is your marital status?	1. Single<br>2. Married<br>3. Divorced<br>4. Widowed
4.	Educational status	____________________
5.	Employee condition?	1. Permanent<br>2. Partime
6.	What is your monthly income?	_____________ETB
7.	How long did you drive? (experience)	_____________Year
8.	Level of your driving license	
9.	How many families do you have?	__________________
**PartII: Drivers factors**		
1.	What type of vehicle does he/she drive?	
2.	Working hours per day?	_____________hrs
3.	Have you taken first aid training?	1. Yes<br>2. No
4.	Do you have life insurance?	1. Yes<br>2. No
5.	Where do you get information about traffic safety? (More than one answers are possible)	1. Media<br>2. Training<br>3. Friends<br>4. Traffic police (specify)___

6.	Have you taken any traffic-related safety training?	1. Yes<br>2. No
7.	If your answer is yes for Q6, when you were engaged in traffic-related safety training?	1. Before starting this job<br>2. After engaged this
**Part III: Risky personal factors**		
1.	Do you use alcohol?	1. Yes<br>2. No
2.	If your answer is yes for Q2 frequency of driving after having alcoholic beverage within 3hrs?	1. Always<br>2. Usually<br>3. Sometimes<br>4. Seldom
3.	Do you chew khat?	1. Yes<br>2. No
4.	If your answer is yes for Q3, frequency of chewing khat?	1. Always<br>2. Usually<br>3. Sometimes<br>4. Seldom
5.	Do you smoke cigarettes?	1. Yes<br>2. No
6.	Do you ever punished by traffic police for disregarding traffic rules with in last year?	1. Yes<br>2. No
7.	If your answer is yes for Q6, frequency of punishment?	___________times
8.	Do you have a habit of checking vehicles?	1. Yes<br>2. No
9.	If your answer is yes in Q8, then do you check it?	1. At the morning<br>2. Throughout the day<br>3. At the end of/evening
10.	Do you use a safety belt?	1. Yes<br>2. No
11.	If your answer is yes for Q10, frequency of seat belt	1. Always

	use?	2. Usually<br>3. Sometimes<br>4. Seldom
12.	Do you have ever driving habits above the recommended speed?	1. Yes<br>2. No
13.	If your answer is yes for Q12, frequency of driving above recommended speed?	1. Always<br>2. Usually<br>3. Sometimes<br>4. Seldom
**Part IV: Vehicles condition and environmental conditions**		
1.	How many years of a vehicle?	______________Years
2.	Type of vehicle you drive?	___________________
3.	Who is the owner of the vehicle?	1. Private<br>2. Employed
4.	Does your vehicle encounter mechanical problems?	1. Yes<br>2. No
5.	In which road types most traffic accident happening?	1. Asphalted<br>2. Not asphalted
6.	In which weather conditions most road traffic accident happening?	1. Rain<br>2. Windy<br>3. Fog/cloudy
7.	Have you ever had traffic accidents on the roads in the last three years?	1. Yes<br>2. No
8.	If your answer is yes for Q7, what is the cause of accidents?	__________________________
3.	If the cause is collision in Q8, what is the type of collision?	1. Head-on collision<br>2. You rear-ended<br>3. You rear-ended a vehicle in front of you
9.	If your answer is yes for Q7, the consequence of the	1. Death

	accident?	2. Injury<br>3. Property damage
10.	If your answer is yes for Q7, injured happened on whom?	1. Passenger<br>2. Pedestrian<br>3. Driver
11.	If your answer is yes for Q7, death happened on whom?	1. Passenger<br>2. Pedestrian
12.	If your answer is yes for Q7, place of accident	1. In the city<br>2. Outside of city
13.	If your answer is yes for Q7, weather conditions during the accident	1. Rain<br>2. Windy<br>3. Fog/cloudy
14	If your answer is yes for Q7, road type where the accident happened?	1. Asphalt<br>2. No asphalt
15	If your answer is yes for Q7, a time when the accident happened?	1. Day<br>2. After noon<br>3. Night<br>4. Midnight
